# Genome-centric insight into metabolically active microbial population in shallow-sea hydrothermal vents

**DOI:** 10.1186/s40168-022-01351-7

**Published:** 2022-10-14

**Authors:** Xiaofeng Chen, Kai Tang, Mu Zhang, Shujing Liu, Mingming Chen, Peiwen Zhan, Wei Fan, Chen-Tung Arthur Chen, Yao Zhang

**Affiliations:** 1 State Key Laboratory of Marine Environmental Science, College of Ocean and Earth Science, Xiamen University, Xiamen, China; 2 Ocean College, Zhejiang University, Zhoushan, China; 3 Institute of Marine Geology and Chemistry, National Sun Yat-Sen University, Taiwan, China

**Keywords:** Geothermal systems, Metagenome-assembled genomes, Metaproteome, Endemicity, Metabolic functions

## Abstract

**Background:**

Geothermal systems have contributed greatly to both our understanding of the functions of extreme life and the evolutionary history of life itself. Shallow-sea hydrothermal systems are ecological intermediates of deep-sea systems and terrestrial springs, harboring unique and complexed ecosystems, which are well-lit and present physicochemical gradients. The microbial communities of deep-sea and terrestrial geothermal systems have been well-studied at the population genome level, yet little is known about the communities inhabiting the shallow-sea hydrothermal systems and how they compare to those inhabiting other geothermal systems.

**Results:**

Here, we used genome-resolved metagenomic and metaproteomic approaches to probe into the genetic potential and protein expression of microorganisms from the shallow-sea vent fluids off Kueishantao Island. The families *Nautiliaceae* and *Campylobacteraceae* within the Epsilonbacteraeota and the *Thiomicrospiraceae* within the Gammaproteobacteria were prevalent in vent fluids over a 3-year sampling period. We successfully reconstructed the in situ metabolic modules of the predominant populations within the Epsilonbacteraeota and Gammaproteobacteria by mapping the metaproteomic data back to metagenome-assembled genomes. Those active bacteria could use the reductive tricarboxylic acid cycle or Calvin-Benson-Bassham cycle for autotrophic carbon fixation, with the ability to use reduced sulfur species, hydrogen or formate as electron donors, and oxygen as a terminal electron acceptor via cytochrome *bd* oxidase or cytochrome *bb3* oxidase. Comparative metagenomic and genomic analyses revealed dramatic differences between submarine and terrestrial geothermal systems, including microbial functional potentials for carbon fixation and energy conversion. Furthermore, shallow-sea hydrothermal systems shared many of the major microbial genera that were first isolated from deep-sea and terrestrial geothermal systems, while deep-sea and terrestrial geothermal systems shared few genera.

**Conclusions:**

The metabolic machinery of the active populations within Epsilonbacteraeota and Gammaproteobacteria at shallow-sea vents can mirror those living at deep-sea vents. With respect to specific taxa and metabolic potentials, the microbial realm in the shallow-sea hydrothermal system presented ecological linkage to both deep-sea and terrestrial geothermal systems.

Video Abstract

**Supplementary Information:**

The online version contains supplementary material available at 10.1186/s40168-022-01351-7.

## Background

Geothermal systems, one of the most chemically and physically extreme environments on our planet, generally occur along active plate margins, in active volcanic regions or near seamounts located in the center of tectonic plates [1]. The discovery of geothermal systems, harboring spectacular communities, has greatly expanded our knowledge and understanding of the habitat range for and the origin of life [2, 3]. Deep-sea alkaline hydrothermal vents [4] and terrestrial hot springs [3] were proposed as locations that may have provided suitable conditions for prebiotic syntheses and acted as potential cradles of the very first life. Shallow-sea hydrothermal vents (depth < 200 m) not only present fluid geochemistry with temperature and redox gradients created by complexed physicochemical steps like those of deep-sea vents, but also experience exposure to sunlight as terrestrial hot springs do, thus harboring unique ecosystems that host a great biodiversity [1]. Recent emerging evidence strongly supports the view that life would have formed in a well-lit environment undergoing wet-dry cycles with a limited amount of water [5–7] and then would have subsequently expanded into the oceanic environment [5]. Investigations of shallow-sea hydrothermal systems, the physicochemical intermediate of terrestrial and deep-sea geothermal systems, should reveal additional clues for the origin or evolution of life. Moreover, it is well known that the heterogeneity and similarity in microbial populations and functional potentials among distinct geothermal systems to date are not completely elucidated.

Compared to their deep-sea counterparts, shallow-sea hydrothermal vents are more accessible and less expensive to sample by scuba divers. Therefore, shallow-sea vents are good sources for studying genetic adaptation in the hostile environments. Previous studies have confirmed high compositional and functional diversity of chemosynthetic ecosystems in diverse shallow-sea hydrothermal systems using sequencing-based metagenomic technologies [8–13]. The shallow-sea vents, like their deep-sea counterparts, may contain a reservoir of physiologically diverse microorganisms, many of which are endemic to submarine hydrothermal systems at the genus level, such as the *Nautilia* and *Caminibacter* within the class Epsilonproteobacteria (reclassified to a new phylum Epsilonbacteraeota; [14]), and the gammaproteobacterial *Thiomicrospiraceae* [15]. Transcriptomic and metaproteomic studies of shallow-sea hydrothermal microbiomes noted potential connections between the metabolic capacity of the microbial population and chemical characteristics of the vent fluids [16, 17]. These studies have focused on characterizing community-wide metabolic networks in shallow-sea hydrothermal vents; however, our knowledge of population-level networks still remains limited, partly due to the absence of cultivated isolates [18]. Genome-resolved metagenomic analysis of microbial communities enables the reconstruction of individual genomes, providing the necessary information to examine how well they adapt to hydrothermal systems. Accordingly, enormous efforts over the past few years have generated a vast number of metagenome-assembled genomes (MAGs) from deep-sea vents and terrestrial hot springs, some of which may represent putative novel species and contain a wealth of information on their metabolic potential [19–24]. Yet, microbial MAGs from shallow-sea hydrothermal systems are still scarce [17]. Ideally, metagenome-assembled and metaproteomic approaches should be combined to identify the microbial population-level metabolic network, thereby bridging the gap between a microbe’s taxonomy and its functional traits.

**Fig. 1:**
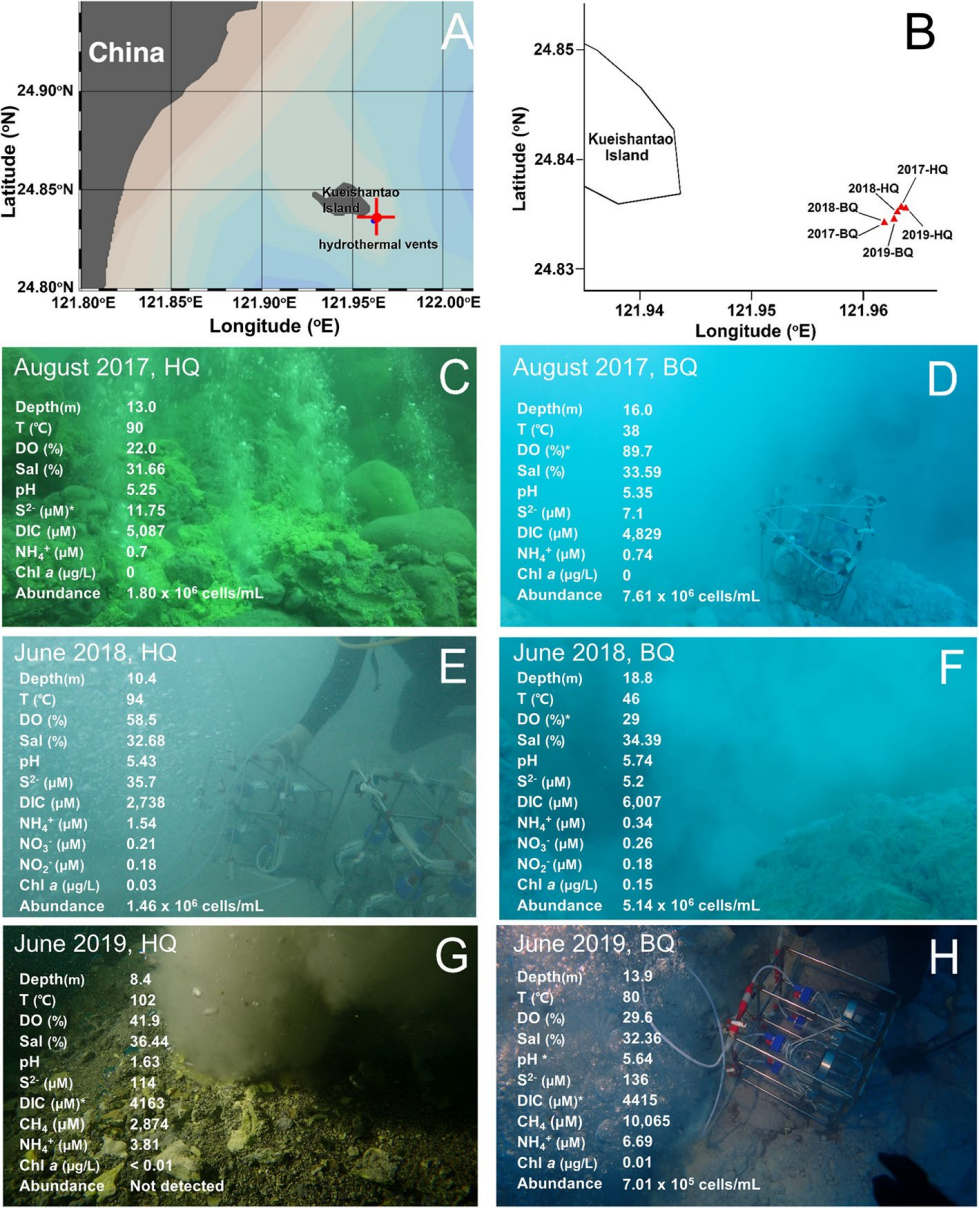
Detail of shallow hydrothermal vents off Kueishantao Island. **A**, **B** Geographical location of hydrothermal vent sampling sites. **C-H** Sample collection at the yellow (HQ) and white (BQ) vents from 2017 to 2019 and the corresponding environmental parameters. T, temperature; DO, dissolved oxygen; Sal, salinity; DIC, dissolved inorganic carbon; Chl *a*, chlorophyll *a*; abundance, cell abundance; asterisk, parameters above the vents

In this study, we sampled the fluids being emitted from vent orifices at an andesite-hosted shallow-sea hydrothermal system off the eastern shore of Kueishantao Island, Taiwan, over a 3-year period (Fig. 1). Metagenomic shotgun sequencing and recovery of MAGs was used to investigate the metabolic function potentials of the collected microorganisms that allow them to survive in the extreme and dynamic vent environment. A MAG-centric metaproteomic approach was used to construct the metabolic modules of those active and predominant populations in the shallow-sea venting fluids. Furthermore, we characterized the functional features of the microbes from geochemically distinct vent fields via metagenomic and genomic analyses. This study not only provides insights into the metabolic machinery of vent-associated microbes living in situ, but also represents a major step forward towards a comprehensive understanding of the biogeographic pattern of microbial metabolic function for different geothermal systems.

## Methods

### Sites and sample collection

Time-series monitoring sampling was performed at two different types of shallow-sea hydrothermal vents, namely yellow vent (HQ) and white vent (BQ), located near Kueishantao Island, offshore of northeast Taiwan, China. The necessary permits had been obtained for the field studies. All samples were taken directly from the vent orifices by scuba divers with the use of polytetrafluoroethylene valves and tubing linked to pre-evacuated glass bottles in August 2017 (HQ, N 24.83554°, E 121.96379°; BQ, N 24.83422°, E 121.96190°), June 2018 (HQ, N 24.83519°, E 121.96303°; BQ, N 24.83418°, E 121.96184°), and June 2019 (HQ, N 24.83560°, E 121.96339°; BQ, N 24.83455°, E 121.96277°), respectively. On board, fluid samples for metagenomic and metaproteomic analyses (10-100 L) were prefiltered through a 3-μm polycarbonate filter (142 mm, Millipore, USA) cutoff, and cells were collected on a 0.2 μm Sterivex filter cartridge (Millipore, USA). After expelling the residual seawater using syringe, 1.8 mL of a sucrose-based lysis buffer (0.75 M sucrose, 40 mM EDTA, 50 mM Tris, pH 8.3) was added to each filter tube, which was then sealed at both ends with parafilm. Filters were placed into 50-mL centrifuge tubes and then frozen by liquid nitrogen immersion and stored at -80 °C until further use. A detailed filtration protocol can be found online at http://www.jove.com/video/1159 [25].

Fluid samples were split into aliquots onboard. Unfiltered fluids were transferred to glass and plastic containers, for carbonate chemistry and nutrient analysis, respectively. Both types of samples were immediately treated with a saturated mercury chloride (HgCl_2_) solution at a volume ratio of 1000:1, and frozen at -20 °C. For prokaryotic abundance, triplicate fluid samples (2 mL, prefiltered by 20 μm) were fixed with glutaraldehyde at a final concentration of 0.5% and stored in the dark at -20 °C. Additional fluid samples (0.5-0.8 L) were filtered using Whatman GF/F glass fiber filters (47 mm), which were stored at -20 °C until the further analysis of chlorophyll *a* (Chl *a*).

### Biogeochemical analysis

The in situ temperature of vent fluids was determined by scuba divers using a thermocouple. Salinity values were measured onboard shortly after collection with an Autosal 8400B salinometer calibrated with IAPSO standard seawater. pH values were measured with a pH meter (Radiometer PHM-85, Denmark) at 25 °C, with a precision better than 0.003 pH unit. Dissolved oxygen was measured using a gas chromatographic method. Nitrate, nitrite, and ammonium nitrogen concentrations were colorimetrically analyzed with a continuous flow autoanalyzer (QuAAtro, SEAL Analytical Inc.). Dissolved sulfide concentrations were assayed by the methylene blue method [26] (the precision was 0.7% at 20 μM, *n* = 6), immediately after returning to the land-based laboratory. Dissolved CH_4_ was measured by gas chromatography using the gas-stripping method [27]. A dissolved inorganic analyzer (AS-C3, Apollo SciTech, United States) was employed for the analysis of dissolved inorganic carbon values, with a precision of 0.1%. Chl *a* was extracted from the filters using the acetone extraction method and measured with a fluorometer according to the procedure summarized in [28]. Prokaryotic abundances were determined with an Accuri C6 flow cytometer (BD Biosciences).

### DNA extraction, metagenomic sequencing, and assembly

Total genomic DNA was extracted from Sterivex filters as described in [29]. Briefly, lysozyme (0.125 mg mL^−1^) was added to the thawed Sterivex filters and then incubated at 37 °C for 1 h with intermittent mixing, followed by the addition of Proteinase K (Qiagen) and 20% SDS and incubated at 55 °C for 2 h with intermittent mixing. Cell lysate was removed using a syringe, and the filters were then rinsed with lysis buffer that was combined with the original lysate. The lysate was extracted by phenol: chloroform: IAA (25:24:1, pH 8.0) and the aqueous layer collected by centrifugation, then loaded onto a 10 K Amicon Ultra filter cartridge (Millipore), washed three times with 2 mL TE buffer, and concentrated by centrifugation to a final volume of 200-500 μL. The concentration and the quality of all DNA samples were evaluated using the NanoDrop-1000 (Thermo Fisher Scientific, Waltham, MA, USA) and agarose gel electrophoresis, respectively. DNA was fragmented via Covaris M220 sonicator (Covaris, Woburn, MA, USA), and the library was prepared with the NEBNext DNA Library Prep Kit (Illumina) following the manufacturer’s instructions. Paired-end (2 × 150 bp) metagenomes were sequenced on the Illumina Hiseq platform. Sequence quality was assessed with FastQC v0.11.2 [30], and sequences were then trimmed using Trimmomatic v0.36 [31] to remove adaptors and low-quality bases. A minimum quality score of 20 and a read length higher than 35 were required. The clean reads were assembled using the assembler IDBA-UD v1.1.2 [32].

### Metagenomic binning and annotation

Genome reconstruction utilizing the metagenomic sequencing data was performed with the function modules of metaWRAP v1.1.1 [33] to recover individual genomes. The module MetaWRAP Bin_refinement was used for further refinement on the sets of bins. Genome quality was estimated using CheckM v1.0.12 [34] to calculate genome completeness and contamination (Table S1). The Metagenome-Assembled Genome standards set up by the Genomic Standards Consortium [35] were also used to evaluate the quality of the MAGs (Table S1).

Open reading frames of the assembled contigs from the metagenomes and MAGS were predicted using Prodigal v2.60 [36] for those sequences longer than 100 bp. The sequences were clustered using CD-HIT [37], with 85% coverage and 90% identity. Gene sets were aligned to NCBI non-redundant database using DIAMOND BLASTP [38] for taxonomic annotations, with an e-value cutoff of 1 × 10^−5^. The predicted protein coding sequences were blasted (BLASTP, e-value < 10^−5^) against eggNOG using DIAMOND to obtain Clusters of Orthologous Groups of proteins (COG) annotation [38, 39]. GhostKOALA servers were used to obtain Kyoto Encyclopedia of Genes and Genomes annotations for the gene set [40]. To determine the relative abundances of predicted genes both for metagenomes and MAGs, Bowtie2 v2.1.0 (local alignment, default settings) was used to perform alignments between the clean reads with the non-redundant gene set [41]. SAMtools v0.1.18 was used to count the number of annotated sequences matched to each gene [42]. Reads per kilobase per million mapped reads (RPKM) calculations were carried out to facilitate transparent comparison of gene abundance between samples [43]. Sequencing and analysis summary statistics for the number of generated reads and the quality of all the metagenomes are provided in Supplementary Table S2.

### Phylogenetic and comparative genomic analysis

Taxonomic lineages of MAGs were classified using the recently developed database GTDB release v95 [44] and the relevant Genome Taxonomy Database Toolkit GTDB-Tk v1.2.0 [45]. A phylogenetic tree using a concatenated alignment of four single-copy orthologous genes shared by all bins (COG0016, COG0532, COG0533, and COG0541) was constructed by the Maximum Likelihood method using MEGA X with LG+F model plus a gamma distribution with eight categories [46]. Bootstrap resampling was performed for 1000 replications. Phylogenetic trees were visualized with Evolview v3 [47]. Comparative genome analyses were carried out on all MAGs in this study, combined with publicly available microbial genomes from marine and terrestrial geothermal systems in the Integrated Microbial Genomes (IMG) database [48]. To retrieve the gene information, searches of the IMG database were performed using both keywords and enzyme codes, and the selected sequences were filtered manually. The average nucleotide identity (ANI) and amino acid identity (AAI) between two genome sequences were calculated by enveomics tools [49]. The previously defined six types (I-VI) of 92 sulfide quinone oxidoreductase (Sqr) amino acid sequences [50] were used as seeds to find *sqr* homologs within the metagenomic and genomic datasets by BLASTP, and a phylogenetic tree of Sqr protein sequences was created by the Neighbor-Joining method using MEGA X for classification of Sqr [46]. Putative hydrogenase classification was confirmed and classified using HydDB [51].

Metagenomes in the Integrated Microbial Genomes and Metagenomes (IMG/M) [48] databases were selected for comparative analysis of microbial community in the geothermal systems on the basis of Illumina sequencing method and assembled sequences (total gene counts > 10,000). A total of 32, 159, and 45 metagenomes derived from shallow-sea, deep-sea hydrothermal systems, and terrestrial hot springs, respectively, were used. These datasets included 20 metagenomes from our previous [18] and current studies on Kueishantao hydrothermal system. A detailed description of the IMG metagenomes can be found in Table S3. We normalized the KO/COG functional profiles to relative cell numbers, dividing the abundance of a given gene by the median abundance of 38 universal single-copy marker genes [52]. The utilized marker genes were K01869, K01872, K01876, K01887, K01889, K01890, K02337, K02338, K02356, K02519, K02520, K02863, K02871, K02874, K02876, K02878, K02879, K02881, K02884, K02886, K02887, K02890, K02895, K02967, K02982, K02986, K02988, K02990, K02992, K02994, K02996, K03070, K03076, K03470, K03550, K03551, K03553, and K03625. One-way analysis of variance (ANOVA) was used to determine the significant differences of parameters among samples.

### Environmental protein extraction and identification

The extraction of total protein from Sterivex filters was performed as described in [29]. Briefly, BugBuster (Novagen) was added to the Sterivex filters to lyse cells, and the lysate was then recovered using a syringe. Buffer exchange was implemented with 100 mM NH_4_HCO_3_ on a 10K Amicon (Millipore). Samples were amended with urea and dithiothreitol and then subject to overnight trypsin digestion, followed by purification with μ-C18 Zip Tips (Millipore) and vacuum dried. The peptides were resuspended in solution A (0.1% formic acid in 2% acetonitrile) and separated by Nano HPLC (Easy-nLC 1000 HPLC system, Thermo Fisher Scientific) then analyzed by tandem mass spectrometry (MS/MS) in a Q Exactive Plus mass spectrometer (Thermo Fisher Scientific). Separation was achieved using a self-packed reversed-phase analytical column (C18, 15-cm length, 75 μm i.d.) with a segmented linear gradient at 350 nL min^−1^: 5-8% solution B (0.1% formic acid in 90% acetonitrile) for 2 min, 8-24% solution B for 40 min, 24-36% solution B for 12 min, 36-80% solution B for 3 min, and finally maintained at 80% solution B for 3 min. The electrospray voltage applied was 2.0 kV. Mass spectrometry data were acquired using a data-dependent procedure with a full scan (350-1600 m/z, resolution 60,000) followed by MS/MS (100 m/z, resolution 15,000; HCD relative collision energy 28%) on the 10 most intense ions with a dynamic exclusion duration of 30 s. The acquired spectral data were searched using the MaxQuant search engine (v.1.5.2.8) [53] to a target-decoy database, containing the peptide sequences of the corresponding metagenome (ORFs counts: 74,611, 48,331, and 673,029 for 2018 BQ and HQ, and 2019 BQ metagenome, respectively), as well as reverse decoy database and common laboratory contaminants. The following parameters were used for protein identification: enzyme type, trypsin; maximum missed cleavage, 2; precursor ion tolerance: 5 ppm; fragment ion tolerance: 0.02 Da; fixed modification: carbamidomethyl cysteine; variable modifications: oxidation on methionine, N-terminal protein acetylation). Minimum score for peptides was set to more than 40 and false discovery rate was adjusted to less than 1%. The normalized spectral abundance factor (NSAF) values were calculated [54], normalizing spectral count values to protein size and to the sum of all spectral counts in each sample, thus giving the relative abundance of a given protein or protein group that could be compared between samples.

## Results

### Environmental parameters

The two shallow-sea vents fields, HQ and BQ, expelled elemental sulfur-rich yellowish and whitish fluids, respectively [16]. The emitted vent fluids were rich in dissolved inorganic carbon (2738-5087 μM), acidic (pH 5-6 on average, lowest recoded pH was 1.6), and hot (temperatures between 90 and 102 °C in HQ, between 38 and 80 °C in BQ) (Fig. 1). The fluids were rich in oxygen and presented high variation in the actual concentrations between distinct sites (Fig. 1). Microbial abundances decreased as temperature increased in the shallow-sea hydrothermal vent environment, ranging from 7.01 × 10^5^ to 7.61 × 10^6^ cells mL^−1^, with cell abundances in BQ higher than in HQ (Fig. 1). In addition, the microbial abundance reached undetectable levels at the HQ site when extremely hot and acidic fluids (102 °C, pH 1.6) were present in 2019 (Fig. 1), showing a natural limit to life in this shallow-sea hydrothermal system. In contrast to the abundance of microbial photosynthetic organisms in terrestrial hot springs, these organisms were scarce in the shallow vents with extremely low or undetectable Chl *a* contents (Fig. 1).

### Metagenomic analysis of shallow vents

**Fig. 2:**
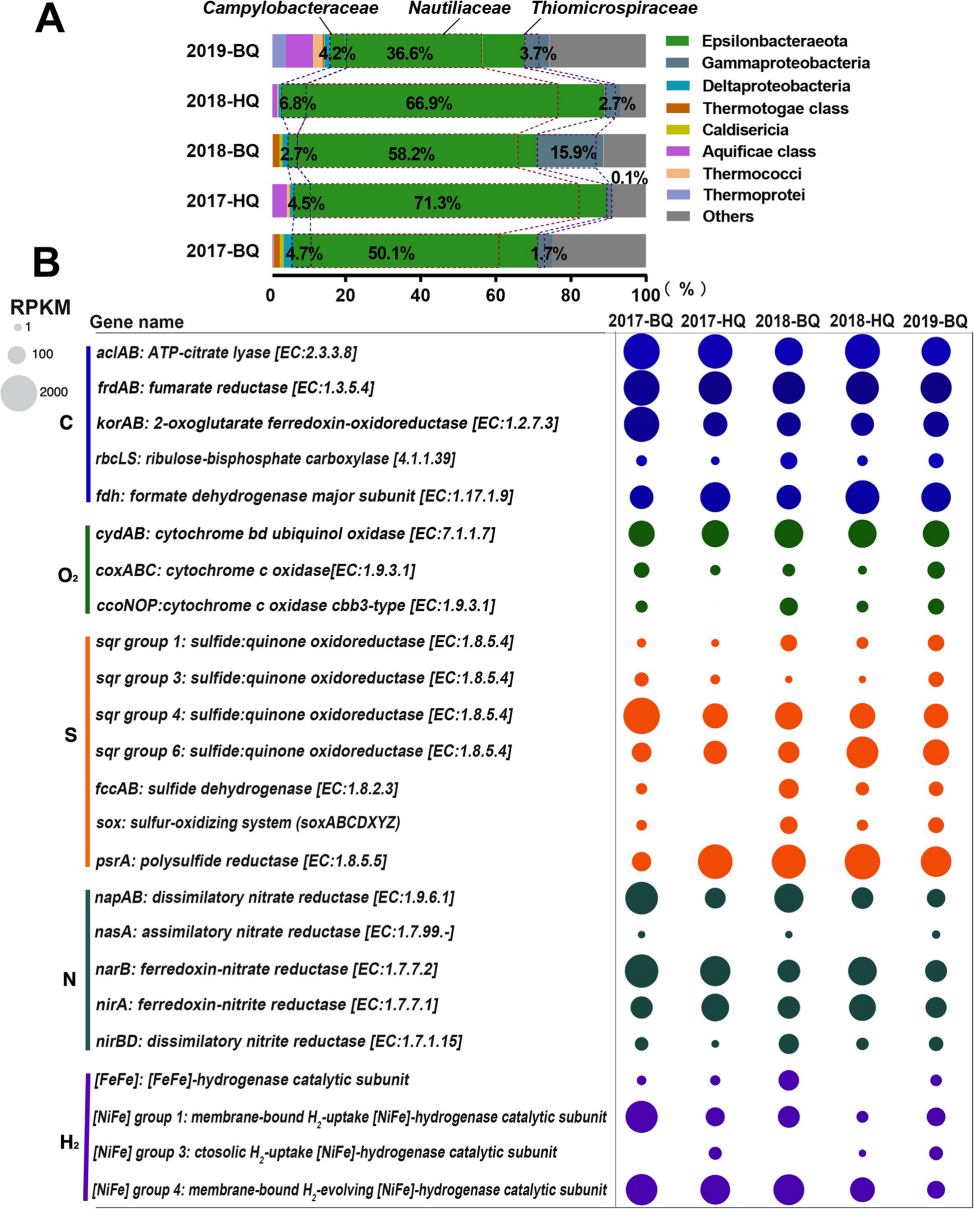
Taxonomic and functional structures of the metagenomes from HQ and BQ for each year. **A** Taxonomy of functional genes in the metagenomes, and the relative abundance of microorganisms at the family level. **B** Metabolic and functional genes involved in carbon fixation (C), terminal oxidase (O_2_), sulfur metabolism (S), nitrogen metabolism (N), and hydrogen oxidation (H_2_). Bubble size indicates reads per kilobase per million mapped (RPKM) for metagenomic reads mapped to the selected genes in each metagenome. For details on functional genes, see supplementary Table S4

The metagenomes of microbial communities over the 3-year period contained highly abundant reads assigned to the genes from Epsilonbacteraeota, of which the majority of matching hits were to *Nautiliaceae* (up to 71.3% of the total functional gene sequences in an HQ metagenomic dataset from 2017), followed by *Campylobacteraceae* (Fig. 2A). The sequences assigned to genes from the gammaproteobacterial *Thiomicrospiraceae* were also prevalent, but their relative abundance varied markedly in the metagenomic datasets (0.1-15.9% of all annotated sequences) (Fig. 2A). Epsilonbacteraeota have the ability to tolerate a much higher sulfide concentration than Gammaproteobacteria, which possibly facilitates their competitiveness in colonizing sulfidic environments [17]. During our sampling timeframe, sequences matching genes from Epsilonbacteraeota occupied a higher percentage in HQ metagenomes than BQ, while those matching genes from *Thiomicrospiraceae* displayed the opposite trend (Fig. 2A), consistent with the higher sulfide concentrations and lower pH in HQ (Fig. 1). Of the archaeal matches, the most common hits were Crenarchaeota (class Thermoprotei) and Euryarchaeota (class Thermococci) (Fig. 2A). They accounted for < 1% of the total assigned sequences in metagenomic datasets from 2017 and 2018, whereas in a BQ metagenomic dataset from 2019, they comprised 4.1% and 2.5% of the total annotated sequences, respectively. Approximately 0.1-1.3% of the total gene sequences were assigned to cyanobacteria in datasets (contained within the “others” group in Fig. 2A).

The relative abundances of genes involved in aerobic respiration, H_2_, carbon, nitrogen, sulfur and phosphorus metabolisms from the metagenomic datasets are available in Table S4. The genes involved in carbon fixation, sulfur metabolism, hydrogen oxidation, oxygen utilization, and denitrification were similar in each metagenome, but their abundances varied (Fig. 2B). The relative abundances of the genes encoding ATP-citrate lyase (*aclAB*, RPKM ranged from 777.22 to 2345.20), as markers for the reductive tricarboxylic acid cycle (rTCA) [55], as well as key genes encoding fumarate reductase (*frdAB*, 881.69-1117.45 RPKM) and 2-oxoglutarate: ferredoxin oxidoreductase (*korAB*, 485.84-1630.51 RPKM) were abundant in the metaproteomes (Table S4). Their relative abundances were higher than those encoding ribulose-1,5-bisphosphate carboxylase (*rbcLS*, < 100 RPKM; Table S4), the markers for the Calvin-Benson-Bassham cycle (CBB) [55]. This result suggested that the rTCA cycle was potentially a major CO_2_ fixation pathway in these shallow-sea hydrothermal vents. Investigation of the genes related to sulfur oxidation showed that type IV and type VI sulfite:quinone oxidoreductase (*sqr*) genes [50] were numerically dominant in all datasets, with RPKM values of up to 2587.85 and 965.77, respectively (Fig. 2B), while genes encoding flavocytochrome *c* sulfide dehydrogenase (*fccAB*), from the truncated sox multienzyme system (*sox*) genes for sulfur oxidation [56], were found at lower abundances among the metagenomic datasets (< 200 RPKM; Fig. 2B and Table S4). FccAB mediates the initial step of H_2_S oxidation to elemental sulfur (S^0^), which has been observed to function under low sulfide conditions [56]. All metagenomes contained a gene encoding for polysulfide reductase (*psr*; Fig. 2B), which is responsible for the quinone-coupled reduction of polysulfide to H_2_ [57]. Genes encoding formate dehydrogenase (*fdh*) for the oxidation of formate [58] were enriched in the metagenomes (473.45-1180.78 RPKM). [NiFe]-hydrogenases-encoding sequences for the oxidation of H_2_ [59] were abundant in the metagenomes (306.44-1915.07 RPKM; Fig. 2B and Table S4). Metagenomes revealed the presence of genes encoding for high-affinity cytochrome *bd*-type (*cydAB*, averaged RPKM of 733.99) and *cbb3*-type oxygen reductases (*ccoNOP*, up to 120.52 RPKM), as well as low-affinity cytochrome *c* oxidases (*coxABC*, less than 100 RPKM) [60]. Genes involved in dissimilatory (*napAB* and *nirBD*) and assimilatory (*narB* and *nirA*) nitrate reduction to ammonium [61] were also present in the metagenomes (Fig. 2B; Table S4). Based on the genes participating in the redox reactions mentioned above, it could be hypothesized that microorganisms from these vent communities had the genetic potential to gain energy using sulfide, hydrogen, and formate as electron donors, while utilizing oxygen, polysulfide and nitrate as electron acceptors.

### Taxonomic profiles of the MAGs

**Fig. 3:**
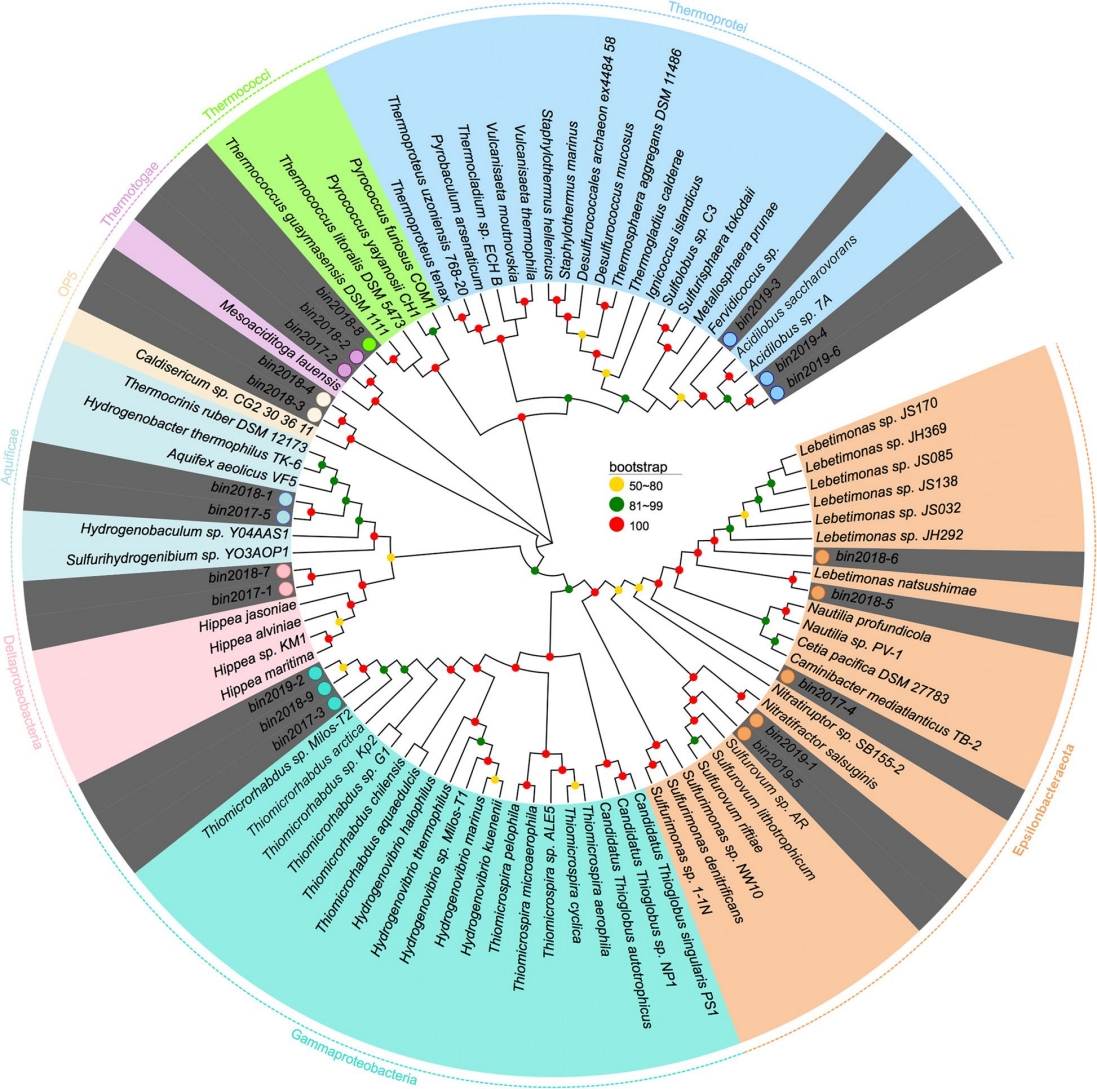
Phylogenetic assignment of the assembled genome bins. The phylogenetic tree was constructed with MEGA using conserved proteins to extract the phylogenetic signal. Organisms are colored by phylum and MAGs are highlighted in gray. Bootstrap node supports ≥ 50 is shown and denoted by a colored dot. For details on genome bins, see supplementary Table S1

As shown in Fig. 3 and Table S1, a total of eleven high-quality (91.1-100% completeness) and nine medium-quality (81.4-89.3% completeness) genome bins were retrieved from the metagenomic data. These MAGs belonged to Epsilonbacteraeota (*n* = 5), Gammaproteobacteria (*n* = 3), Deltaproteobacteria (*n* = 2), Aquificae (*n* = 2), Caldiserica (*n* = 2), Thermotogae (*n* = 2), Thermococci (*n* = 1), and Thermoprotei (*n* = 3) (Table S1). The Epsilonbacteraeota MAGs belonged to the families *Nautiliaceae* and *Sulfurovaceae*, which could be assigned to the genera *Lebetimonas* (bins 2018-5 and 2018-6), *Nitratifractor* (bin 2019-1), and *Sulfurovum* (bin 2019-5), as they shared 84.5%, 90.9%, and 86.9% genome-aggregate ANI, and 89.5%, 92.6%, and 87.6% genome-aggregate AAI with their closest relatives, respectively (Table S5). Epsilonbacteraeota bin 2017-4 had low ANI (73.6%) and AAI (62%) values with *Nautilia profundicola* (Table S5). Three gammaproteobacterial MAGs (bins 2017-3, 2018-9, and 2019-2) were placed within the family *Thiomicrospiraceae* and shared a 100% ANI with each other, thus representing the same species. They shared 79.7% ANI and 79.4% AAI with *Thiomicrorhabdus* sp. Kp2 (Table S5). Similarly, the deltaproteobacterial MAGs (bins 2017-1 and 2018-7) and Aquificae MAGs (bins 2017-5 and 2018-1) were assigned to the same species, respectively, based on their respective intra-group ANI values of 99.9% and 99.3% (Table S5).

Each sample was binned individually, allowing us to evaluate the abundance and persistence of the recovered MAGs over the course of 3 years using metagenome fragment recruitment against the MAGs. One *Nautiliaceae* MAG (bin 2017-4) represented the most prevalent population accounting for approximately 2.32-11.57% in all metagenomes (Fig. S1). The two *Lebetimonas* MAGs (bins 2018-5 and 2018-6) accounted for approximately 13.41% and 2.63% of the BQ metagenomes reads in 2018, respectively, but they were only a small fraction (< 1%) in other samples (Fig. S1). The *Thiomicrorhabdus* MAGs (bins 2017-3, 2018-9, and 2019-2) were consistently observed each year and contributed up to approximately 12.78% of the BQ metagenome reads in 2018 (Fig. S1). Therefore, *Nautiliaceae* and *Thiomicrospiraceae* MAGs represented a stable core of the indigenous bacterial populations in these vent fluids. The abundances of the remaining recovered MAGs were below 1% for most of the samples. Of the archaeal MAGs, Thermoprotei (bins 2019-3, 2019-4, and 2019-6) were only detected at very low abundances (approximately 0.06% and 0.12%) of the 2019 BQ metagenome and the abundance of Thermococci (bin 2018-8) was only approximately 0.01% of both the 2018 HQ and 2019 BQ metagenomes (Fig. S1).

### Metaproteomic analysis of shallow-vent-associated microbes

**Fig. 4:**
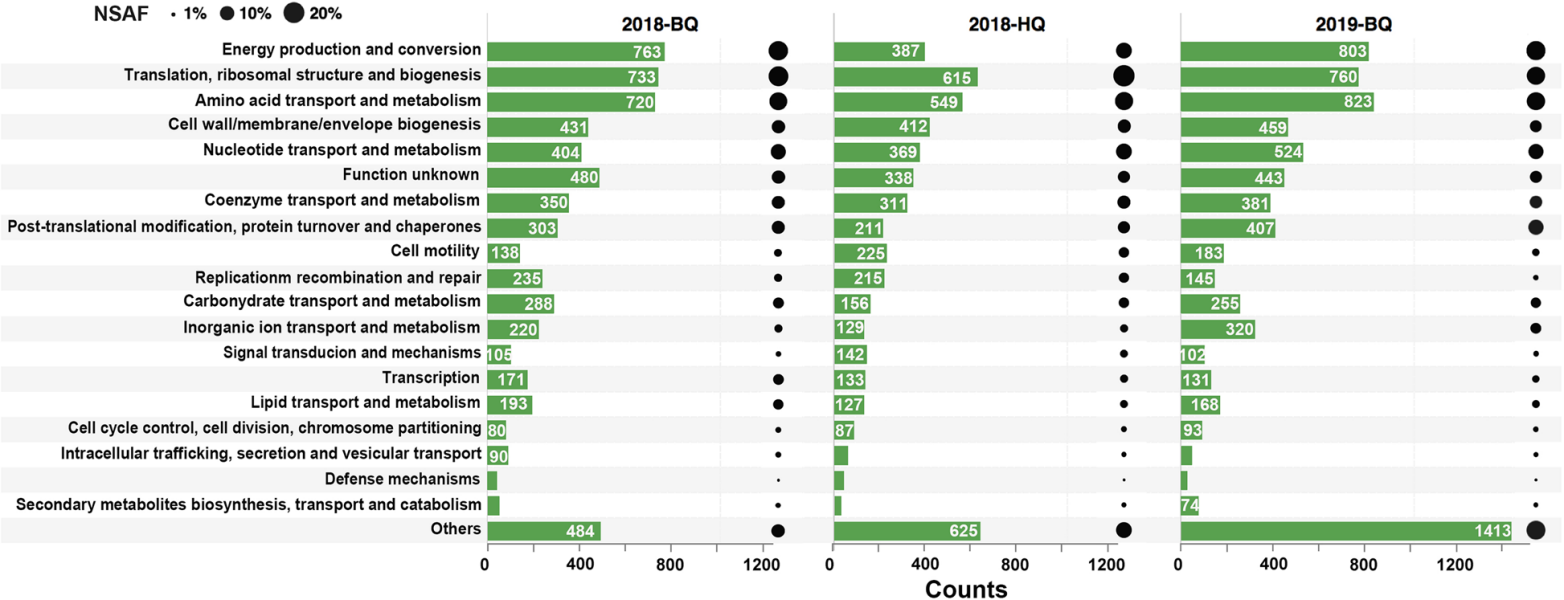
Protein distribution in COGs. Proteins identified in the metaproteomic data cover all major COG categories. The bars represent the number of proteins within each COG category and the numbers are also displayed. The non-COG categories are classified as “Others”. The circles representing NSAF values provide relative peptide abundances in each category. For details of proteomic data, see supplementary Table S6

A metaproteomic approach provides insight into the in situ expression of metabolic pathways of microbial communities at a given point in time. We performed protein mass spectrometry of samples from BQ and HQ in 2018 and BQ in 2019, yielding 23,680, 14,464, and 21,260 unique peptides, corresponding to 6286, 5164, and 7559 protein groups, respectively (Table S6). At the COG category level, proteins involved in energy production, translation, amino acid, and nucleotide metabolism as well as cell wall and membrane biogenesis, together, accounted for at least 50% of the relative abundances of peptides per metaproteome (Fig. 4). Meanwhile, proteins associated with carbohydrate metabolism were expressed at abundances of only 3-4% (Fig. 4). In addition, transporter proteins were present in the vent metaproteomes at approximately 1%, 5%, and 9% of total identified proteins in the 2018 HQ, 2018 BQ and 2019 BQ samples, respectively (Fig. S2), while those previously found in metaproteomes of surface and deep seawater heterotrophic communities generally account for 23-39% [62]. The diverse organic nutrient transporters that are generally rich in heterotrophic ecosystems, such as transporters for carbohydrates (including monosaccharides, disaccharides, and oligosaccharides), dicarboxylates, taurine, and urea [62], were not detected in the 2018 HQ metaproteome (Table S6).

The homologous proteins assigned to the recovered 20 MAGs, together, occupied to 77%, 59%, and 59% of total detected proteins in 2018 BQ and HQ, and 2019 BQ metaproteome, respectively. The information of homologous proteins assigned to each MAG in metaproteomes is shown in Table S7. The majority of identified proteins were affiliated with the Epsilonbacteraeota, followed by Gammaproteobacteria, which is in line with the observation in metagenomes. The organism with the highest overall abundance in the metaproteomes was *Nautiliaceae* bin 2017-4, accounting for 29% of the total identified proteins on average. *Lebetimonas* MAGs (on average 19% and 14% of metaproteomes for bins 2018-5 and 2018-6, respectively) and *Thiomicrorhabdus* MAGs (13% of metaproteome on average) were highly expressed as well. These data indicated that these bacterial populations represented the core and active taxa of the microbial communities, whose metabolic functions would reflect how the resident organisms could tolerate a hydrothermal environment.

### Reconstruction of metabolic modules of predominant and active shallow-vent populations

**Fig. 5:**
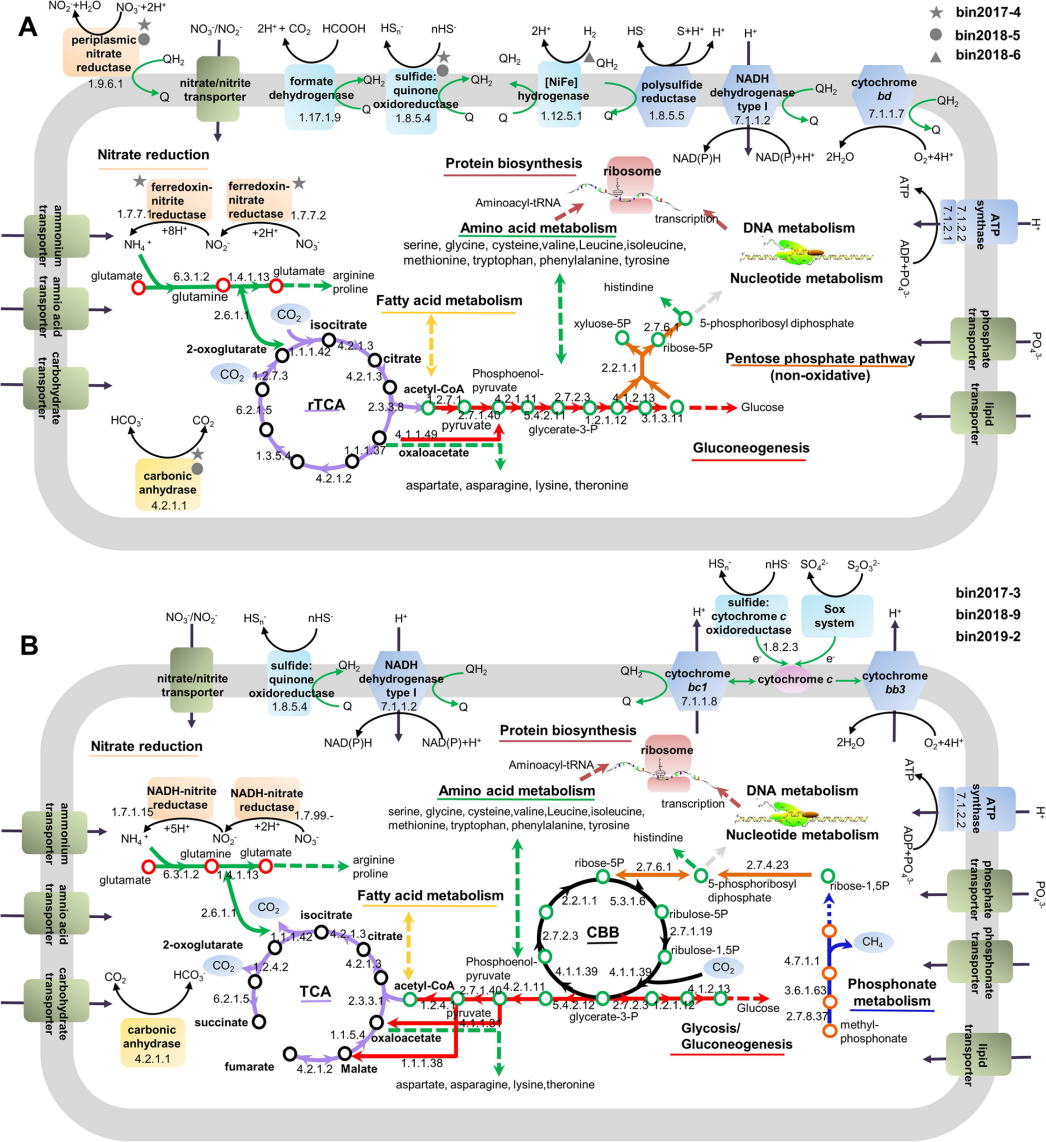
Reconstructed in situ metabolic pathways of the recovered bins within Epsilonbacteraeota and Gammaproteobacteria based on proteins detected in the metaproteomic data. **A** Metabolic pathways of Epsilonbacteraeota bins 2017-4, 2018-5, and 2018-6, all proteins were detected in all three bins, except those marked by star, circle, and triangle. **B** Metabolic pathways of gammaproteobacterial bins 2017-3, 2018-9, and 2019-2. Selected metabolic processes and other cellular activities are represented by the name of the enzyme or the substrate of the enzyme. Enzymes were identified by searching against selected organisms (Table S8). Continuous lines represent complete metabolic processes for which all the relevant proteins were detected in the metaproteomes, while dashed lines represent pathways where only some of the proteins involved were detected (Table S8). Q, ubiquinone; QH_2_, ubiquinol

Based on the protein profiles of individual MAGs, mapped back to their respective genomic data, we reconstructed and examined the core metabolic functions responsible for adaption to thermally and geochemically dynamic habitats. All enzymes required for a complete rTCA cycle and gluconeogenesis, as well as phosphoenolpyruvate carboxykinase for the removal of intermediates from the rTCA cycle [63], were expressed in Epsilonbacteraeota bins 2017-4, 2018-5 and 2018-6 (Fig. 5A and Table S8). Enzymes for the non-oxidative pentose phosphate pathway that synthesizes ribose 5-phosphate for use in nucleotide biosynthesis [64] were also expressed (Fig. 5A and Table S8). *Nautiliaceae* bin 2017-4 and *Lebetimonas* bin 2018-5 expressed Sqr for the oxidation of H_2_S, which would provide energy for their chemolithoautotrophic growth (Fig. 5A). *Lebetimonas* bin 2018-6 lacked Sqr, but possessed a [NiFe] hydrogenase (Fig. 5A), enabling it to use H_2_ as an energy source. All three Epsilonbacteraeota MAGs had the ability to use formate as electron donors in the presence of formate dehydrogenase (Fig. 5A). They could use the cytochrome *bd* complex for oxidizing ubiquinol-reducing oxygen as part of the aerobic respiratory electron transport chain (Fig. 5A). Their type I NAD(P)H dehydrogenases [65] could contribute to electron transfer either through forward electron transfer (NAD(P)H to ubiquinone) or reverse electron transfer (ubiquinol to NAD^+^) (Fig. 5A). The presence of nitrate reductase and polysulfide reductase (Psr) would allow all three Epsilonbacteraeota MAGs to use nitrate and polysulfide as the respective electron acceptors (Fig. 5A). Psr might conserve energy by proton translocation while balancing the reduced quinone pool [57]. The expression of multiple energetic metabolic pathways simultaneously for chemosynthesis may enable these populations to flourish across diverse redox gradients.

All enzymes involved in the CBB cycle and gluconeogenesis/glycolysis were identified in the gammaproteobacterial *Thiomicrorhabdus* MAGs (Fig. 5B and Table S8). Succinate dehydrogenase that catalyzes the oxidation of succinate into fumarate [66] was absent in these MAGs, indicating an incomplete TCA cycle (Fig. 5B). These MAGs expressed malic enzyme and phosphoenolpyruvate carboxylase for the entry of intermediates (pyruvate and phosphoenolpyruvate, respectively) into the TCA cycle [67] (Fig. 5B), ensuring their ability to convert metabolites from the CBB cycle to biosynthetic precursors as well as generate reducing equivalents. Besides Sqr, their protein profiles revealed that the oxidation of reduced sulfur compounds could proceed via the FccAB and Sox pathways (Fig. 5B). Together, these two pathways are proposed to produce energy and reducing power via a reverse and forward electron transfer of sulfur oxidation, including cytochrome *c* proteins, cytochrome *bc1* complex, and type I NADH dehydrogenase (or NADPH)-quinone oxidoreductases (Fig. 5B). Proteomic data indicated that these *Thiomicrorhabdus* respired oxygen with a cytochrome *bb3* terminal oxidase (Fig. 5B and Table S8). The metaproteomic results indicated that these MAGs had the ability to couple chemosynthesis with multiple energy metabolisms.

Two Epsilonbacteraeota (bins 2017-4 and 2018-5) and all three gammaproteobacterial MAGs could express carbonic anhydrase enzyme to sequester CO_2_ [68], which could be subsequently incorporated into organic material (Table S8). Proteins involved in assimilatory nitrate reduction to ammonium were present in *Nautiliaceae* bin 2017-4 and all of the gammaproteobacterial *Thiomicrorhabdus* MAGs (Fig. 5). Further, glutamine synthetase for the assimilation of ammonium [69] was found in all these microorganisms (Fig. 5). The results indicated that these microorganisms are capable of meeting their nitrogen requirement via endogenous synthesis, whereas heterotrophs showed high expression of proteins for the uptake of organic nitrogen substrates [70]. Additionally, *Thiomicrorhabdus* microorganisms could use methylphosphonic acid as a phosphorus source if the transporter and enzymes involved in methylphosphonate utilization were present [71] (Fig. 5B). There were between 59 and 77 transporter genes in the Epsilonbacteraeota MAGs, less than the number observed in previously sequenced heterotrophic Epsilonbacteraeota (75 to 124 genes) [70]. The autotrophic *Thiomicrorhabdus* MAGs possessed 87 to 113 genes coding for transporters, fewer than the number commonly found in heterotrophic gammaproteobacterial genomes (approximately 110 to 250 genes). Additionally, approximately 25.3-59.2% of the total transporter genes in these MAGs were expressed (Fig. S3), while generally 54.0-85.4% of the total transporter genes could be expressed in heterotrophic bacteria [72]. This is an apparent difference between these autotrophs and heterotrophs.

### Metagenomic and genomic comparison of microbes from geothermal systems

**Fig. 6:**
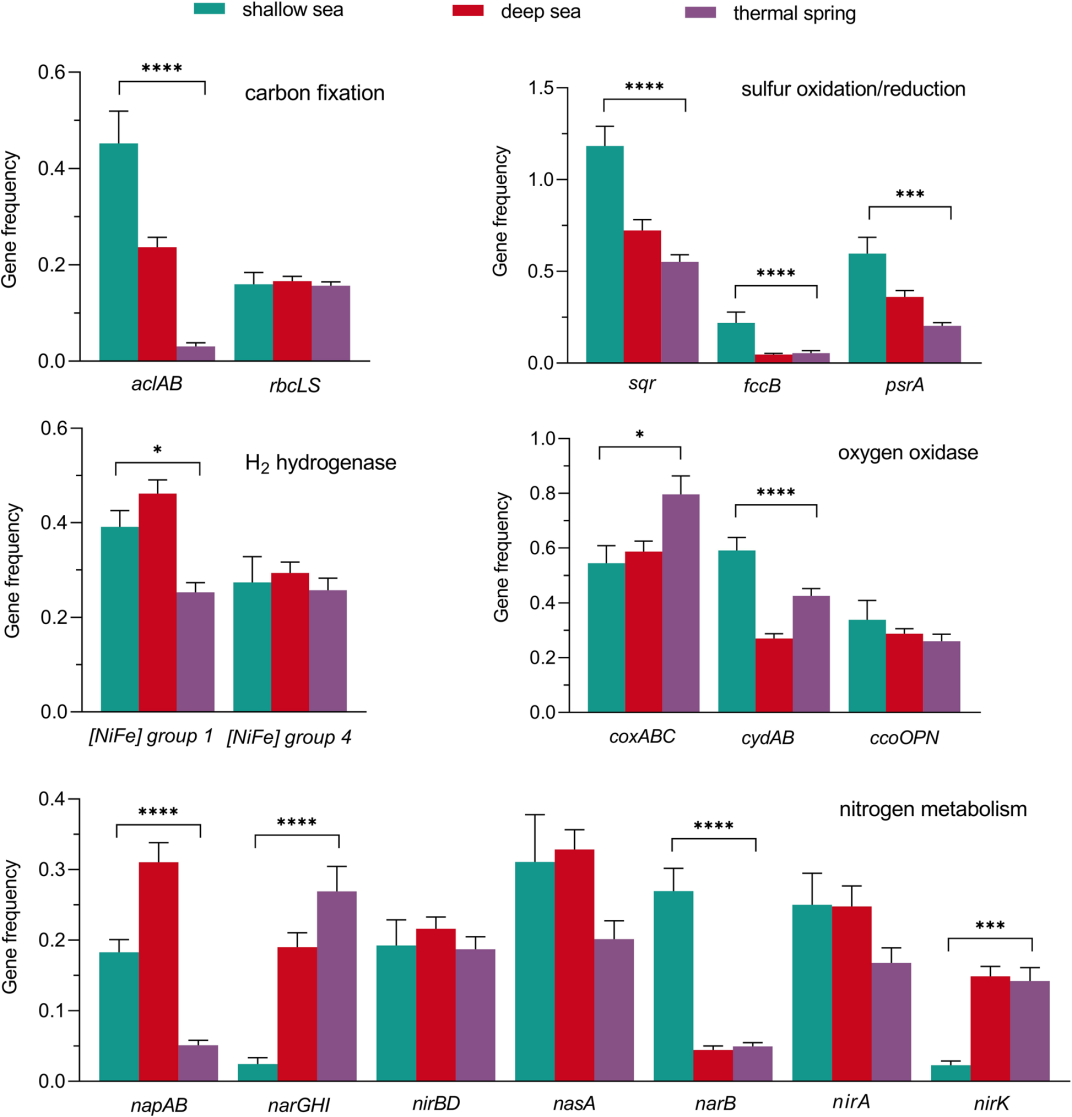
The gene frequency of key metabolic genes in the metagenomes of shallow-sea, deep-sea, and terrestrial geothermal systems. More detail on datasets is shown in Table S3. The average gene frequencies are presented as mean values ± standard error of mean. Differences were considered significant if the *p*-value of an ANOVA was less than 0.05 (*, *p* < 0.05; ***, *p* < 0.001; ****, *p* < 0.0001). Green, shallow-sea hydrothermal systems; red, deep-sea hydrothermal systems; purple, thermal spring

**Fig. 7:**
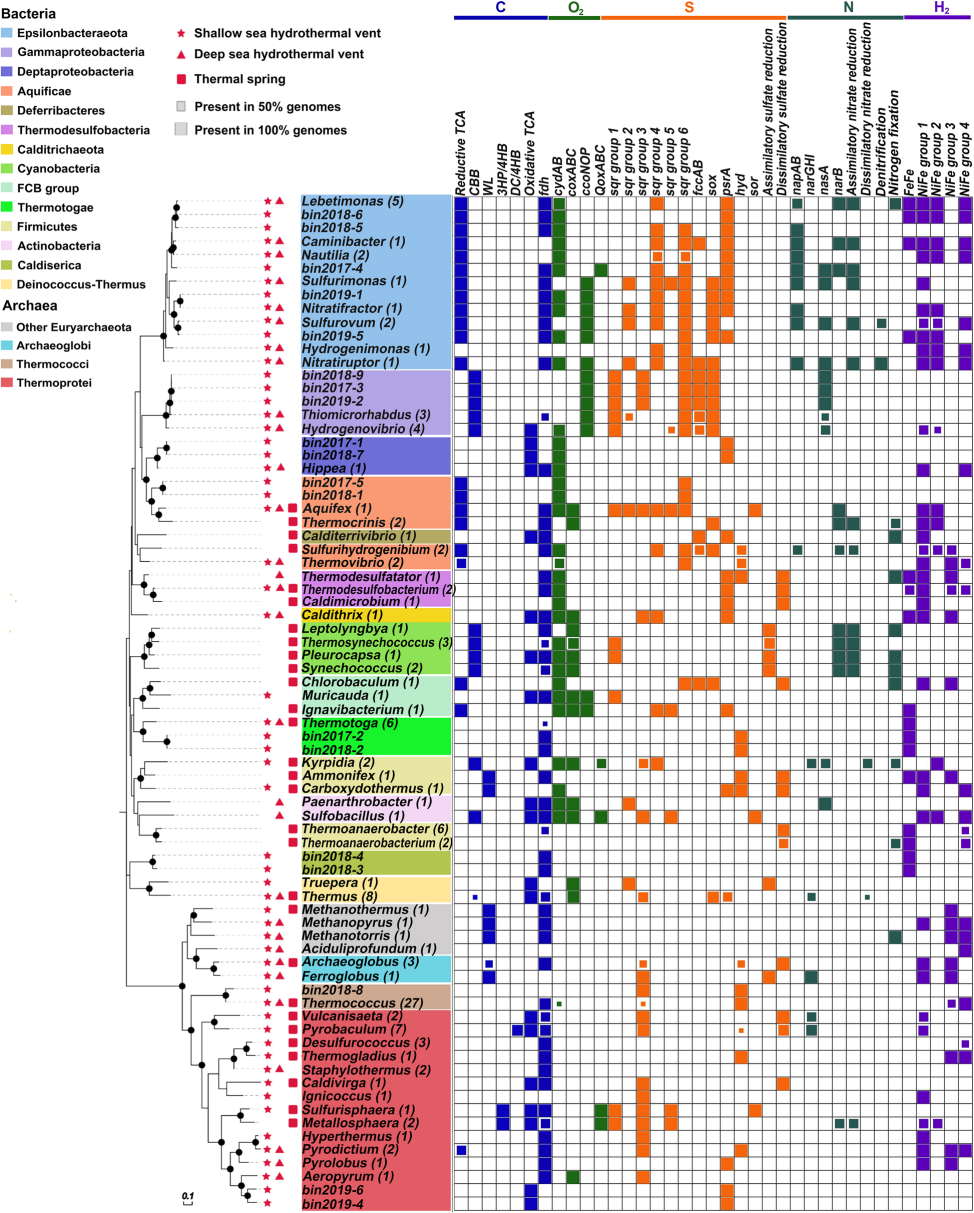
Distribution of metabolic modules and genes in bacterial and archaeal genomes derived from marine hydrothermal systems and terrestrial hot springs. The available genomes of cultured organisms were downloaded from the IMG database and were searched against metagenomic studies to finally obtain the habitat of specific microbial genus. Organisms are colored by taxonomic classification (see legend). The metabolic and functional genes included carbon metabolism (C), terminal oxidase (O_2_), sulfur metabolism (S), nitrogen metabolism (N), and hydrogen oxidation (H_2_). Habitat types of strains are indicated by different shapes, and the number of sequenced complete genomes is shown in brackets. Genes encode the complete enzyme and all enzymes of the pathway, respectively, show the presence of corresponding functions and pathways in the genome. The frequency of occurrence of various functions of interest for each genus is shown with squares of proportional size. Bootstrap node supports ≥ 50 are denoted by a black dot, the scale bar at the bottom indicates 20% sequence divergence. *CBB*, Calvin-Benson-Bassham cycle; *WL*, Wood-Ljungdahl pathway; *3HP/4HB*, 3-hydroxypropionate/4-hydroxybutyrate; *DC/4HB*, dicarboxylate/4-hydroxybutyrate cycle; *fdh*, formate dehydrogenase; *cydAB*, cytochrome *bd* quinol oxidase; *ccoNOP*, cytochrome *cbb3* oxidase; *coxABC*, *aa3*-type cytochrome *c* oxidase; *QoxABC*, the ubiquinol-dependent cytochrome *ba3* oxidase; *sqr*, sulfite:quinone oxidoreductase; *fccAB*, flavocytochrome *c* sulfide dehydrogenase; *sox*, sox multienzyme system; *psrA*, polysulfide reductase subunit A; *hyd,* sulfhydrogenase; *sor*, sulfur oxygenase/reductase; *napAB*, dissimilatory nitrate reductase; *narGHI*, dissimilatory nitrate reductase; *nasA*, assimilatory nitrate reductase catalytic subunit; *narB,* ferredoxin-nitrate reductase. For details on functional genes, see supplementary Tables S9 and S10

We performed an in-depth comparative genomic analysis, using both metagenomes and genomes of microbes originating from three types of geothermal systems. The *aclAB* genes were enriched in microbial communities living in marine hydrothermal systems, especially for shallow-sea hydrothermal systems (average of 0.45 genes per cell; Fig. 6). The gene abundances of *alcAB* in deep-sea hydrothermal systems varied in a wide range, with the highest abundance up to 1.27 genes per cell in microbial communities from the East Pacific Rise, Pacific Ocean (Table S3). In contrast, terrestrial hot spring-associated microbial communities possessed a significantly lower *aclAB* abundance (average of 0.03 genes per cell, *p* < 0.0001; Fig. 6). In addition to Epsilonbacteraeota, the rTCA cycle has recently been identified as the CO_2_ fixation mechanism used by members of Aquificae, which are often the dominant species in high-temperature and near-neutral terrestrial hot springs [73]. Consistently, all Epsilonbacteraeota and Aquifex MAGs recovered in this study possessed genes involved in the rTCA cycle (Fig. 7). Microbial *rbcLS* genes did not reveal any significant differences among distinct hydrothermal systems (Fig. 6). The CBB cycle ensures carbon fixation not only in autotrophic Gammaproteobacteria but also in thermophilic cyanobacteria (Fig. 7).

The average gene frequencies of *sqr*, *fccB*, and *psrA* genes were significantly more abundant in shallow-sea hydrothermal systems than other geothermal systems (*p* < 0.001; Fig. 6). The genes encoding [NiFe] hydrogenase group 1 were more abundant in oceanic sites than terrestrial ones (*p* < 0.05; Fig. 6). Furthermore, many Epsilonbacteraeota and Gammaproteobacteria, both those that have been cultured and the MAGs recovered in this study, harbored multiple *sqr* gene families (Fig. 7). Within the culturable genera from submarine vents and terrestrial springs, more than two-thirds of which possess *sqr* genes, while more than half contain multiple sets of hydrogenases (Fig. 7 and Table S9), indicating the importance of sulfide and H_2_ oxidation for energy generation. Notably, most hydrogen utilizers also possess genes encoding Sqr, both for the culturable genera and MAGs recovered in this study (Fig. 7), indicating their ability to generate energy via multiple redox reactions. Additionally, formate oxidation, which is commonly coupled with H_2_ evolution mediated by H_2_-evolving hydrogenases for energy [59], appears to be prevalent in vent- or spring-inhabiting chemoautotrophs, including all of the Epsilonbacteraeota MAGs recovered from the Kueishantao vents (Fig. 7 and Table S10). With respect to aerobic respiratory, microbial communities living in shallow-sea hydrothermal systems were enriched in *cydAB* genes, with the abundance of average 0.59 genes per cell (Fig. 6). While the low-affinity cytochrome *c* oxidase genes *coxABC* were enriched in microbes inhabiting terrestrial springs (averaged 0.54 genes per cell; Fig. 6), where oxygen is easily available due to the rapid atmospheric exchange and products from photosynthesis release [74]. A majority of aerobic autotrophs possesses multiple oxidases genes, including three Epsilonbacteraeota MAGs found in this study (Fig. 7), which could facilitate their growth under different oxygen tensions in a wide range of ecological niches within geothermal systems. Genes encoding the high-affinity nitrate reductase (*napAB*) presented a significantly higher abundance in oceanic than terrestrial geothermal systems (averaging 0.18, 0.31, and 0.05 genes per cell for shallow-sea, deep-sea and terrestrial systems, respectively, *p* < 0.0001; Fig. 6), and were prevalent in Epsilonbacteraeota (Fig. 7). Genes *narGHI* and nitrite reductase gene (*nirK*) displayed significantly lower abundances in shallow-sea hydrothermal microbial communities than other geothermal systems, whereas the ferredoxin-nitrate reductase coding gene (*narB*) was more abundant in shallow-sea hydrothermal systems (*p* < 0.0001; Fig. 6). Though gene *nasA* was also present in shallow-sea hydrothermal systems (Fig. 6), only rare sequences of this gene have been found in the Kueishantao hydrothermal vent sites (Fig. 2B). The complete denitrification and dissimilatory reduction of nitrate appeared to be uncommon among prokaryotes from geothermal systems (Fig. 7).

## Discussion

### Endemicity of microbial populations in shallow-sea hydrothermal systems

Hydrothermal systems present a variety of ecological niches enabling the colonization of diverse microorganisms, many of which are endemic to these systems. A total of 20 of the 57 culturable genera had a restricted distribution, appearing only in one category of geothermal system (Table S10). Shallow-sea hydrothermal systems shared a total of 28 genera (15 bacterial and 13 archaeal) and 14 genera (5 bacterial and 9 archaeal) with deep-sea hydrothermal systems and terrestrial hot springs, respectively (Fig. 7). While only 5 genera (3 bacterial and 2 archaeal) were common to deep-sea hydrothermal systems and terrestrial hot springs, which were also found in shallow-sea hydrothermal systems (Fig. 7). Thermophilic cyanobacteria are endemic to terrestrial hot springs (Fig. 7) and have successfully colonized this extreme environment [75]. Culturable genera of Epsilonbacteraeota and Gammaproteobacterial isolates have been retrieved from marine hydrothermal systems; however, none of those genera have been observed in terrestrial hot springs (Fig. 7), suggesting a high degree of endemicity in marine hydrothermal vents.

Members of the *Nautiliaceae* within Epsilonbacteraeota and *Thiomicrospiraceae* within Gammaproteobacteria have frequently been found to be the major active bacterial groups in the hydrothermal systems of Kueishantao Island [9, 16, 76]. Here we consistently recovered MAGs belonging to *Nautiliaceae* and *Thiomicrospiraceae* during our 3-year investigation period and successfully resolved their in situ protein expression at the population level for the first time. The genera of *Nautiliaceae* and *Thiomicrospiraceae* are also prevalent at deep-sea hydrothermal vents; however, their metabolic processes operating in deep-sea vents in situ at the individual population level are poorly described [77, 78]. Cultured *Nautiliaceae* species can grow autotrophically via the rTCA cycle for carbon fixation, using S^0^ reduction coupled with the oxidation of H_2_S, H_2_, or formate [79], and these processes were found to operate in situ in their relatives thriving at the Kueishantao vents (Fig. 5A). *Thiomicrorhabdus* MAGs within *Thiomicrospiraceae* possessed the potential ability to generate energy via the oxidation of multiple reduced sulfur compounds and transfer electrons to oxygen or nitrate (Fig. 5B). This result is in line with previous studies of deep-sea vent-associated *Thiomicrorhabdus* species which noted their metabolic versatility as aerobic chemoautotrophs [80–82]. Thus, the metabolic machinery of the active and predominant *Nautiliaceae* and *Thiomicrospiraceae* populations at shallow-sea vents can mirror those living at deep-sea vents.

### Linking microbial community functional potential to shallow-sea hydrothermal environmental settings

#### Autotrophy

The physical and chemical characteristics of geothermal systems play a significant role in constraining microbial functional metabolisms. Phototrophs could inhabit not only terrestrial hot springs but also shallow-sea hydrothermal systems where sunlight is present, including the Kueishantao system [9, 76], which represents a striking difference with deep-sea vent systems [1]. Generally, the rTCA cycle is the major pathway for chemosynthesis in marine hydrothermal systems, including the Kueishantao vents (Figs. 2 and 6). However, the in situ chemosynthetic carbon fixation rates suggested only a minor contribution to the primary production of terrestrial hot spring ecosystem, accounting for approximately 3% of the local photosynthetic carbon fixation rates [83]. Many autotrophic hydrothermal microbes, similar to the gammaproteobacterial *Thiomicrorhabdus*, appear to have an incomplete oxidative TCA cycle, including but not limited to members of Thermodesulfobacteria and methanogens (Fig. 7). Generating energy chemosynthetically from reduced chemicals, instead of by the complete oxidation of exogenous organic carbon, might give an advantage to the living hydrothermal microorganisms to thrive in such extreme environments. The primary function of the incomplete TCA cycle might be responsible for producing metabolites for growth [84], reflecting adaptation to life in the vents. In contrast to a heterotrophic community, with its high expression of proteins involved in carbohydrate metabolism and organic matter transporter functions [60, 70, 72], the chemolithoautotrophs-dominant Kueishantao community could meet their growth requirements mainly via endogenous synthesis.

#### Energy conversion

Geothermal fluids often contain reduced sulfur species (mainly H_2_S and S^0^), though the actual composition and concentration can vary dramatically depending on the geological setting [85, 86]. The andesite-hosted Kueishantao hydrothermal system contained μM level concentrations of H_2_S and were rich in S^0^ [87]. The metagenomic and metaproteomic results revealed that oxidation of reduced sulfur species, especially H_2_S, constituted the most abundant chemolithotrophic energy metabolism in all Kueishantao hydrothermal vents (Tables S4 and S6). Fluids from shallow-sea hydrothermal vents are often characterized by low H_2_ concentrations, as compared to that of deep-sea hydrothermal vents, and trace H_2_ was found in Kueishantao hydrothermal fluids [87, 88]. The Kueishantao vent metagenomes contained a much lower proportion of putative hydrogenase sequences compared to those from H_2_-rich environments (Table S3), such as serpentinizing deep-sea hydrothermal systems [89]. Nevertheless, hydrogenases were actively expressed in situ by microbes inhabiting this hydrothermal system (Table S6), enabling them to utilize the available H_2_ for energy generation.

Geothermal autotrophs that possess the capability of aerobic respiration have a competitive advantage in microbial communities, due to the greater energy yields from the oxidation of reducing substrates with oxygen compared to nitrate or sulfate respiration [90]. It is noteworthy that the uncultured *Nautiliaceae* members recovered within this study could utilize O_2_ as an electron acceptor (Fig. 5A) and were persistently abundant in the O_2_-rich Kueishantao hydrothermal fluids (Fig. 2A), while all cultured *Nautiliaceae* species could only grow under anaerobic conditions [79]. Given that a bacterial phenotype is the result of multiple factors, there would be distinctions in observations between laboratory or shipboard incubations and in situ processes. The culturable species of *Nautiliaceae* that possessed *cydAB* genes (Fig. 7), may present a tolerance for oxygen in submarine hydrothermal systems. Furthermore, the *bd*-type oxidase were the most abundant terminal oxidases in the metagenomes and metaproteomes of all Kueishantao vent colonizing microbial communities (Tables S4 and S6), suggesting their ability to grow in even low-oxygen habitats as well as maintain redox balance [91]. Under anaerobic or microaerobic conditions of geothermal habitats, several microorganisms that possess nitrate or nitrite reductase genes, mainly Epsilonbacteraeota and Gammaproteobacteria (Fig. 7), could utilize nitrate or nitrite as an electron acceptor. Epsilonbacteraeota could simultaneously express nitrate reductase and cytochrome oxidases, such as in the prevalent *Nautiliaceae* populations of the Kueishantao vents (Fig. 5A), which may enable them to flourish across diverse redox gradients. High rates of sulfate reduction are frequently measured in terrestrial hot springs, enhanced by the presence of photosynthesis products [92–94], whereas the sulfate reduction rates at marine hydrothermal systems are much lower [95, 96]. Although sulfate is plentiful in Kueishantao hydrothermal fluids, with concentrations of approximately 24.5-30.4 mM [87], only a small number of sulfate-reducing bacteria [16] and few gene sequences involved in sulfate reduction (this study; Table S4) were present in the Kueishantao bacterial community.

The functional potentials analyses results suggested the metabolic versatility of microbial communities as chemolithoautotrophs in the Kueishantao shallow-sea vents, generating energy via multiple redox reactions for adapting to low H_2_S, H_2_-poor, and O_2_-rich environments.

#### Rare taxa of shallow-sea vents as future research hotspots

The steep geochemical (oxic to anoxic) gradients at shallow-sea vents not only contains aerobic microorganisms but also harbors anaerobic ones that occupy a narrow range of ecological niches and may include rare populations within Thermotogae, Caldiserica, Thermococci, and Thermoprotei (Fig. 7). These taxa could serve as a reservoir of special functional potentials, like that in deep-sea vents [97, 98]. It was recently shown that high CO_2_ levels drive the TCA cycle backward (namely reversed oxidative tricarboxylic acid cycle) [99], allowing carbon fixation in bacteria inhabiting geothermal systems, such as the hot spring-associated *Desulfurella acetivorans* [100], deep-sea vent-associated *Thermosulfidibacter takaii* [101], and shallow-sea hydrothermal-associated *Hippea martima* [102]. Such a carbon fixation strategy was thought to operate in microorganisms on CO_2_-rich ancient Earth [102]. Here, we found that autotrophs from Kueishantao Island submarine vents harbored a similar capability for carbon fixation. For example, two bins (2017-1 and 2018-7) belonging to the thermophilic anaerobic bacterial genus *Hippea* (Fig. 7). Additionally, shallow-sea vents contain special and rare species, including thermophilic nitrogen fixing bacteria (such as *Methanotorris*), a radiation-resistant bacterium (in *Truepera*) [103], the only currently known hyperthermophilic archaeal host (in *Ignicoccus*) [104] and a hyperthermophilic and neutrophilic archaeon (in *Hyperthermu*s) [105] (Fig. 7). These results indicated that the rare taxa of shallow-sea hydrothermal systems might curate a seed bank of functional genes involved in ancient metabolic pathways that have survived from when such microbes throve in the early anoxic Earth’s geothermal environments. Hence, shallow-sea hydrothermal rare taxa are worth further exploration to expand our knowledge of microbial metabolic functions in extreme environments and provide clues about the microbial life of early Earth.

## Conclusions

Here, genome-centric metagenomic analysis of vent-associated microbiomes over a 3-year period allowed the recovery of 20 MAGs, substantially increasing the number of genomes sequenced from shallow-sea hydrothermal environments. The metabolic modules of the active and predominant populations within Epsilonbacteraeota and Gammaproteobacteria in shallow-sea vents could mirror those living in deep-sea vents, as suggested by the metagenomic and metaproteomic data. Although hydrothermal microbiomes exhibit different facets of functional traits including the adaption to regional environmental conditions, the microbial functional signatures in the shallow-sea hydrothermal system shed light on a linkage to deep-sea and terrestrial counterparts. Future investigations on the intrinsic functions of rare species from shallow-sea hydrothermal systems could reveal undiscovered metabolic capabilities and facilitate our understanding of microbial ecology and evolution in extreme environments.

## Supplementary Information

**Additional file 1: Fig. S1.** Fragment recruitment analysis. **Fig. S2.** The relative abundance of transporter proteins in metaproteome. **Fig. S3.** Profiles of transporter genes in the assembled MAGs within Epsilonbacteraeota and Gammaproteobacteria.

**Additional file 2: Table S1.** Genome statistics for MAGs.

**Additional file 3: Table S2.** Summary of the metagenome sequencing statistics.

**Additional file 4: Table S3.** Features and functional gene counts of metagenomic data sets from geothermal systems used for comparison analysis.

**Additional file 5: Table S4.** Relative abundance (reads per kilobase per million mapped reads, RPKM) of selected key genes in metagenomic datasets.

**Additional file 6: Table S5.** The average nucleotide identity (ANI) and amino acid identity (AAI) between MAGs and their closes relatives.

**Additional file 7: Table S6.** Proteins identified from microbes at shallow-vents and their functional annotations and abundances.

**Additional file 8: Table S7.** Genomic and proteomic information of 20 MAGs recovered in this study.

**Additional file 9: Table S8.** Genomic and proteomic data of the predominant *Nautiliaceae* and *Thiomicrorhabdus* MAGs used for metabolic modules reconstruction.

**Additional file 10: Table S9.** The number of different types of hydrogenase and sulfide quinone oxidoreductase (*sqr*) in microbial genomes.

**Additional file 11: Table S10.** Distributions of selected key enzymes in microbial genomes.

## Data Availability

The metagenome and metaproteome datasets supporting the conclusions of this article are available in the National Omics Data Encyclopedia (NODE, https://www.biosino.org/node/) database with the identifier OEP001339. The metagenomic datasets are identified with the accession numbers OER103520, OER090181, OER090184, OER090179, and OER090185 for 2017-BQ, 2017-HQ, 2018-BQ, 2018-HQ, and 2019-BQ, respectively. The metaproteomic raw data as well as analysis data are available on NODE with the identifiers OER090178, OER090177, and OER090176 for 2018-BQ, 2018-HQ, and 2019-BQ, respectively. The binning MAGs are available through NODE under the accession numbers OED565920 for bin 2017-1, OED565921 for bin 2017-2, OED565924 for bin 2017-3, OED565928 for bin 2017-4, OED565929 for bin 2017-5, OED565932 for bin 2018-1, OED565933 for bin 2018-2, OED565934 for bin 2018-3, OED565938 for bin 2018-4, OED565939 for bin 2018-5, OED565973 for bin 2018-6, OED565975 for bin 2018-7, OED565976 for bin 2018-8, OED565985 for bin 2018-9, OED565988 for bin 2019-1, OED565941 for bin 2019-2, OED565943 for bin 2019-3, OED565944 for bin 2019-4, OED565948 for bin 2019-5, and OED565949 for bin 2019-6, respectively.

## Abbreviations

MAG: Metagenome-assembled genome
HQ: Yellow vent
BQ: White vent
COG: Clusters of Orthologous Groups of proteins
IMG: The Integrated Microbial Genomes database
ANI: Average nucleotide identity
AAI: Amino acid identity
RPKM: Reads per kilobase per million mapped reads
Sqr: Sulfide quinone oxidoreductase
NSAF: The normalized spectral abundance factor
TCA: The tricarboxylic acid cycle
rTCA: The reductive tricarboxylic acid cycle
CBB: The Calvin-Benson-Bassham cycle
Psr: Polysulfide reductase
