# Supplementary material for: Genome-centric insight into metabolically active microbial population in shallow-sea hydrothermal vents
Source: Microbiome. 2022 Oct 14;10:170. doi: 10.1186/s40168-022-01351-7 (PMC9563475; doi:10.1186/s40168-022-01351-7)
Supplement: Supplementary file 2 — Additional file 1: Fig. S1. Fragment recruitment analysis. Fig. S2. The relative abundance of transporter proteins in metaproteome. Fig. S3. Profiles of transporter genes in the assembled MAGs within Epsilonbacteraeota and Gammaproteobacteria. [file 40168_2022_1351_MOESM1_ESM.docx]

**Fig. S1** Fragment recruitment analysis. Recruitment was calculated as the number of reads aligned to MAGs normalized by that of total reads in given metagenomic sample. Color code represents the relative abundance of reads aligned to MAGs in each metagenome. The average nucleotide identity between bin 2017-3, bin 2018-9 and bin 2019-2 were 100% and their amino acid identify 99.8-100% (Table S5), thus, these MAGs were considered together for calculation.


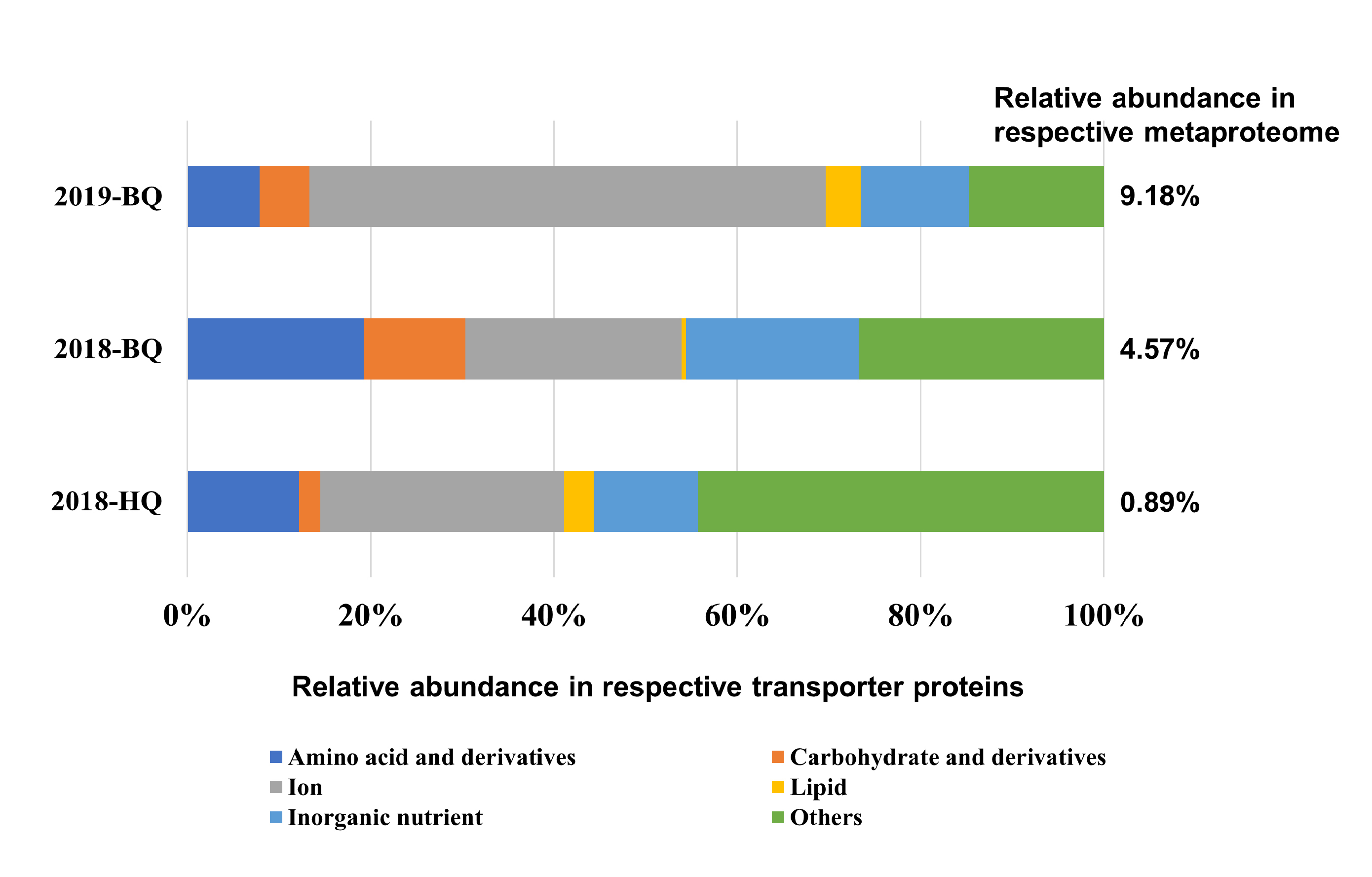


**Fig. S2** The relative abundance of transporter proteins in metaproteome.


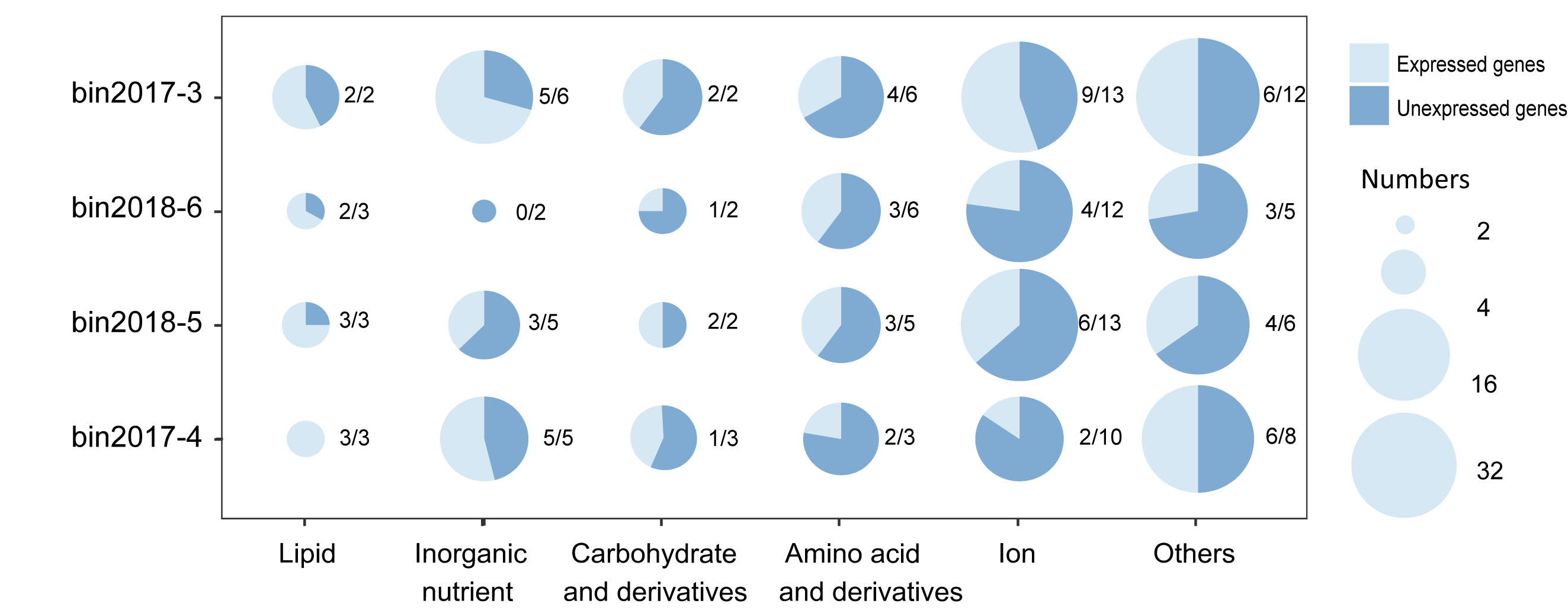


**Fig. S3** Profiles of transporter genes in the assembled MAGs within Epsilonbacteraeota and Gammaproteobacteria. The size of the circle represents the counts of transporter genes. The light blue sections indicate the counts of transporters genes for specific substrates at each MAG. The darker blue sections indicate the counts of expressed transporter genes. The specific values next to circles indicate the number of types of specific transporters: expressed types/ the total types.
